# Evaluating the strengths and limitations of multimodal ChatGPT-4 in detecting glaucoma using fundus images

**DOI:** 10.3389/fopht.2024.1387190

**Published:** 2024-06-07

**Authors:** Saif Aldeen AlRyalat, Ayman Mohammed Musleh, Malik Y. Kahook

**Affiliations:** ^1^ Department of Ophthalmology, The University of Jordan, Amman, Jordan; ^2^ Department of Ophthalmology, Houston Methodist Hospital, Houston, TX, United States; ^3^ Jordan University Hospital, Amman, Jordan; ^4^ Department of Ophthalmology, University of Colorado School of Medicine, Sue Anschutz-Rodgers Eye Center, Aurora, CO, United States

**Keywords:** large language models, glaucoma, artificial intelligence, ChatGPT, GPT

## Abstract

**Overview:**

This study evaluates the diagnostic accuracy of a multimodal large language model (LLM), ChatGPT-4, in recognizing glaucoma using color fundus photographs (CFPs) with a benchmark dataset and without prior training or fine tuning.

**Methods:**

The publicly accessible Retinal Fundus Glaucoma Challenge “REFUGE” dataset was utilized for analyses. The input data consisted of the entire 400 image testing set. The task involved classifying fundus images into either ‘Likely Glaucomatous’ or ‘Likely Non-Glaucomatous’. We constructed a confusion matrix to visualize the results of predictions from ChatGPT-4, focusing on accuracy of binary classifications (glaucoma vs non-glaucoma).

**Results:**

ChatGPT-4 demonstrated an accuracy of 90% with a 95% confidence interval (CI) of 87.06%-92.94%. The sensitivity was found to be 50% (95% CI: 34.51%-65.49%), while the specificity was 94.44% (95% CI: 92.08%-96.81%). The precision was recorded at 50% (95% CI: 34.51%-65.49%), and the F1 Score was 0.50.

**Conclusion:**

ChatGPT-4 achieved relatively high diagnostic accuracy without prior fine tuning on CFPs. Considering the scarcity of data in specialized medical fields, including ophthalmology, the use of advanced AI techniques, such as LLMs, might require less data for training compared to other forms of AI with potential savings in time and financial resources. It may also pave the way for the development of innovative tools to support specialized medical care, particularly those dependent on multimodal data for diagnosis and follow-up, irrespective of resource constraints.

## 1 Introduction

Medical applications of artificial intelligence (AI) have been constantly evolving over the past decades. This is particularly true of machine learning (ML), deep learning (DL), and eventually the emergence of large language models (LLM) (1). Among the first medical applications in AI was a glaucoma model for patient consultation (2). Most recently, the emergence of LLMs represented a breakthrough that disrupted existing models. Transfer learning with high quality foundational models was needed to reach a certain accuracy. With the advancement of computational models, less data were needed to achieve high accuracy output data with potential for clinical utility.

Earlier AI models demanded large datasets to achieve noteworthy accuracy, posing a challenge in the era of data scarcity. However, the landscape began to shift as advancements in ML and DL algorithms allowed for the development of models capable of achieving remarkable accuracy with smaller datasets, harnessing methods of transfer learning (1). This evolution marked a critical juncture, enabling the integration of AI into medical applications with a reduced dependence on extensive data sources. Of the fields that witnessed such evolution was ophthalmology, where a model for glaucoma consultation was among the first to be developed in late 1970s (2). Since then, AI research in glaucoma in the form of peer reviewed publications has expanded exponentially (3). While early attempts focused on specific tasks of pattern recognition and basic image analysis (4), but the true potential of AI in healthcare began to unfold with the advent of large-scale language models (5). ChatGPT is a publicly available LLM available in multiple versions, including ChatGPT3.5 and ChatGPT-4. While ChatGPT3.5 is a text-based platform and freely accessible, ChatGPT-4 is a multimodal model, able to accept input data in the form of text or images and requires a subscription for access. In this study, we aimed to evaluate the diagnostic accuracy of the multimodal ChatGPT-4 in recognizing glaucoma using color fundus photography (CFP).

## 2 Methods

### 2.1 Description of datasets

**Figure 1:**
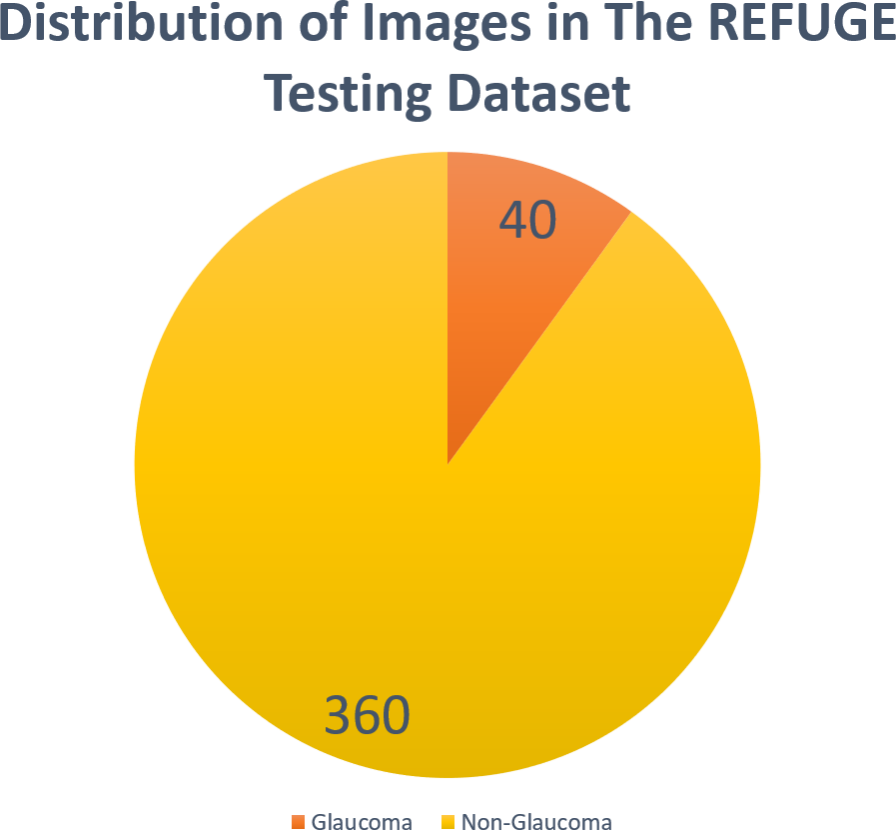
Distribution of images in the REFUGE testing dataset.

We used the publicly accessible retinal fundus glaucoma challenge (REFUGE) dataset (6). REFUGE consists of a collection of 1200 CFPs, divided into three equal subsets of training, validation, and testing sets, each containing 400 images, in JPEG format, from Chinese patients obtained from various hospitals and clinical studies. The images are centered on the posterior pole to display the optic nerve head (ONH). The dataset comprises 10% of images that exhibit glaucoma characteristics and includes patients diagnosed with two types of glaucoma: primary open angle glaucoma (POAG) and normal tension glaucoma (NTG). Glaucomatous cases were identified based on ONH damage and reproducible visual field defects. Non-glaucomatous images from healthy individuals as well as patients with myopia, diabetic retinopathy, and megalopapilae are also included. The overall dataset utilized in this study was divided into 90% of non-glaucomatous images and 10% of glaucomatous images (**Figure 1**).

### 2.2 Prompt design

We adopted a method described by Lyu et al. (7), which has demonstrated better performance from ChatGPT (OpenAI: https://chat.openai.com/) when presented with the following prompt: “Please design the best prompt for you based on this prompt,” followed by a specific task description. We applied this methodology to ChatGPT-4, requesting it to generate an optimal prompt based on a detailed task description, with minor edits by authors to further refine its performance.

The prompt:

“Hello ChatGPT, you are simulating an ophthalmologist with a specialization in glaucoma detection using fundus photographs. Your task is to perform a preliminary analysis of the attached fundus photographs to determine whether they show signs of Glaucoma. You are required to classify each photograph as either ‘Likely Glaucomatous’ or ‘Likely Non-Glaucomatous’ based on observable features.

Instructions:

1. Examine each attached fundus photograph, focusing primarily on the ONH and the peripapillary area.
2. For each image, decide if it is:
a. Likely Glaucomatous: Identify characteristic signs of glaucoma such as increased cup-to-disc ratio, thinning of the neuroretinal rim, optic disc hemorrhages, or other glaucomatous optic neuropathy indicators.
b. Likely Non-Glaucomatous: Determine the absence of glaucomatous features, indicating a non-glaucomatous optic nerve head and retinal nerve fiber layer.
3. Provide a definitive classification for each image as either ‘Likely Glaucomatous’ or ‘Likely Non-Glaucomatous’. Refrain from giving uncertain or ambiguous classifications.”

### 2.3 ChatGPT-4 instructions and setup

For analysis, we inputted the entire 400 image of the testing set. The task involved classifying fundus images into either ‘Likely Glaucomatous’ or ‘Likely Non-Glaucomatous’. Initial experimentations involved presenting four images simultaneously to ChatGPT-4 for evaluation. However, inconsistencies in response led us to revise our strategy, proceeding with a single-image analysis. The prompt was tailored to match the characteristics of fundus images in the dataset used to identify glaucoma, ensuring consistency in the diagnostic approach. Each image was presented to ChatGPT-4 individually along with the prompt, and its diagnostic accuracy was compared against the labels provided. Image analysis was conducted between November 24, 2023, and November 28, 2023. Examples of ChatGPT-4 responses can be found in the **Supplementary Material**.

In addition to our primary analysis conducted without image preprocessing, we also performed exploratory experimentations with half of the images to assess the impact of various preprocessing strategies on the performance of ChatGPT-4. This subset comprised the first 200 images from the dataset. We tested two preprocessing techniques including contrast limited adaptive histogram equalization (CLAHE) for contrast enhancement and cropping to focus on the optic disc and the peripapillary area and provided the model with a variation of different number of images per prompt instead of one per prompt.

### 2.4 Performance appraisal

**Figure 2:**
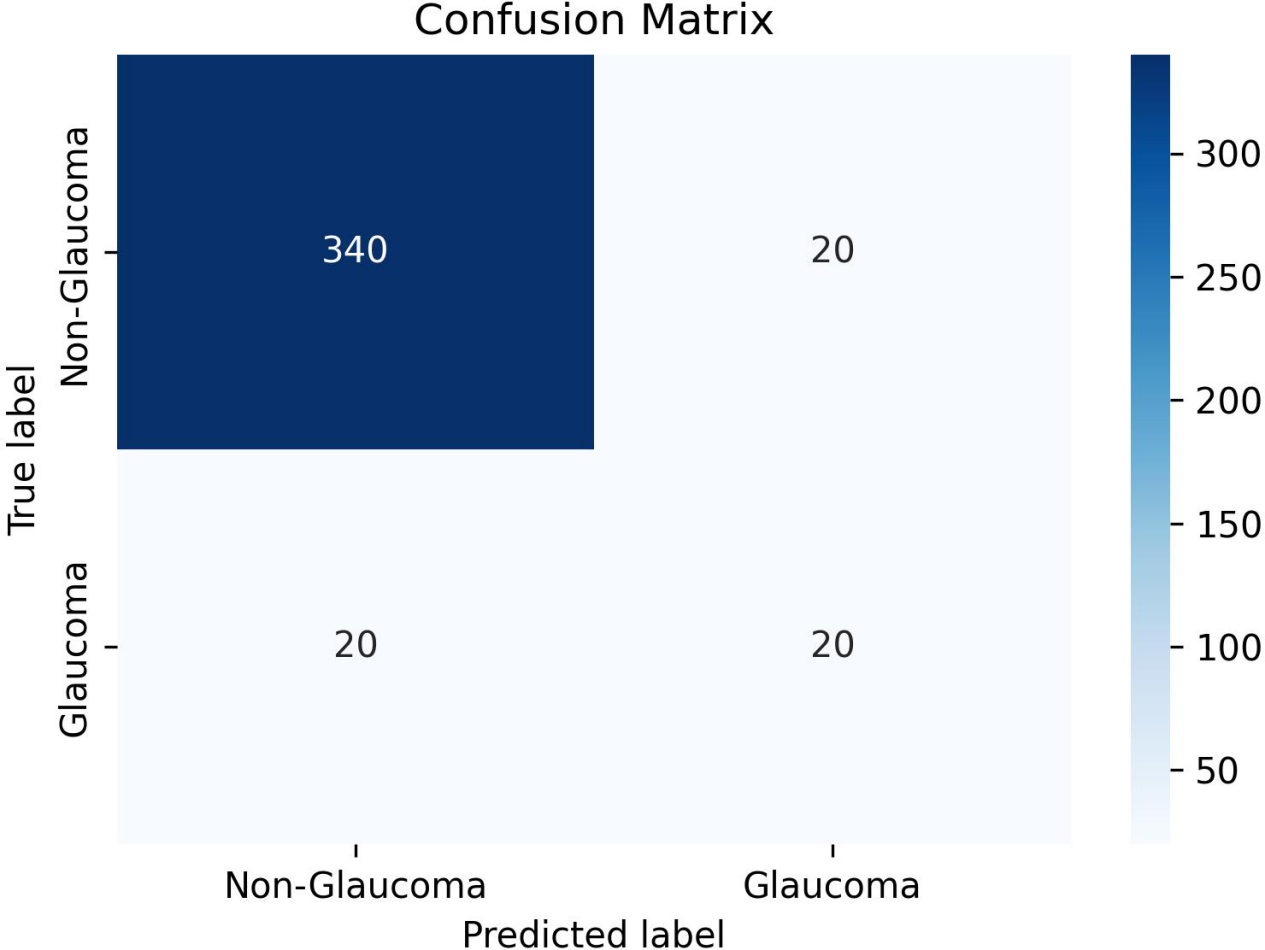
Confusion matrix for binary classification.

We constructed a confusion matrix to visualize the results of ChatGPT-4’s first responses for each image, focusing on binary classifications (glaucoma vs non-glaucoma), as shown in (**Figure 2**). Evaluation metrics included accuracy (Acc), sensitivity (Sen), specificity (Spe), precision (Pre), and F1 score, along with their 95% confidence intervals, when possible, Equations 1–5. All calculations were performed using python script in Google Colab (https://colab.google), and the code used can be found in the **Supplementary Material**.


(1) $$\text{Accuracy}:\frac{\left(TP+TN\right)}{\left(TP+TN+FP+FN\right)}$$

(2) $$\text{Sensitivity }\left(Recall\right):\frac{TP}{\left(TP+FN\right)}$$

(3) $$\text{Specificity}:\frac{TN}{\left(TN+FP\right)}$$

(4) $$\text{Precision}:\frac{TP}{\left(TP+FP\right)}$$

(5) $$\text{F}1\text{ Score}:2*\frac{\left(Precision*Recall\right)}{\left(Precision+Recall\right)}$$

### 2.5 Literature search for comparison

To identify studies that involved binary glaucoma/non-glaucoma classification task using the REFUGE dataset and compare it to ChatGPT-4 performance in terms of accuracy, we searched databases of PubMed, Scopus and Web of science for studies published in English up to 28 November 2023, using the following keywords: “Glaucoma”, “Artificial intelligence”, “Machine Learning”, “Deep Learning”, “REFUGE”, “Retinal Fundus Glaucoma Challenge”. also, a Google Scholar search was performed to identify relevant articles.

## 3 Results

ChatGPT-4 demonstrated an accuracy of 90% with a 95% confidence interval (CI) of 87.06%-92.94%. The sensitivity was found to be 50% (95% CI: 34.51%-65.49%), while the specificity was 94.44% (95% CI: 92.08%-96.81%). **Table 1** shows the results of glaucoma classification by ChatGPT-4. The precision was recorded at 50% (95% CI: 34.51%-65.49%), and the F1 Score was 0.50.Full results of ChatGPT-4 in classifying each image are found in the **Supplementary Table**, in which “0” refers to non-glaucoma images, and “1” refers to glaucoma images.

**Table 1:** Results of binary glaucoma/non-glaucoma classification by ChatGPT-4.

Model	Dataset	Total number of Images	*Acc% (95% CI)*	*Sen% (95% CI)*	*Spe% (95% CI)*	*Pre% (95% CI)*	*F1 score*
ChatGPT-4	REFUGE (Test set)	400	90.00% (95% CI: 87.06%-92.94%)	50.00% (95% CI: 34.51%-65.49%)	94.44% (95% CI: 92.08%-96.81%)	50.00% (95% CI: 34.51%-65.49%)	0.50

After cropping the fundus images to focus solely on the optic disc and peripapillary area, the model achieved a sensitivity of 87.50%. Although this was conducted on a smaller set of images, cropping significantly enhanced the sensitivity of glaucoma detection, correctly identifying 9 images previously misclassified without cropping. However, the specificity was reduced to 56.52%.

Applying CLAHE to the cropped images further improved sensitivity to 62.50%. Despite this, CLAHE, like cropping, resulted in a reduced specificity of 55.43%. **Tables 2**, **3** show the results of glaucoma classification by ChatGPT-4 after preprocessing.

**Table 2:** Results of binary glaucoma/non-glaucoma classification by ChatGPT-4 after Cropping.

Model	Dataset	Total number of Images	*Acc% (95% CI)*	*Sen% (95% CI)*	*Spe% (95% CI)*	*Pre% (95% CI)*	*F1 score*
ChatGPT-4	REFUGE (Test set)	200	59.00% (95% CI: 52.18%-65.82%)	87.50% (95% CI: 71.30%-100.00%)	56.52% (95% CI: 49.36%-63.68%)	14.89% (95% CI: 35.87%-97.46%)	0.25

**Table 3:** Results of binary glaucoma/non-glaucoma classification by ChatGPT-4 after Cropping + CLAHE.

Model	Dataset	Total number of Images	*Acc% (95% CI)*	*Sen% (95% CI)*	*Spe% (95% CI)*	*Pre% (95% CI)*	*F1 score*
ChatGPT-4	REFUGE (Test set)	200	56.00% (95% CI: 49.12%-62.88%)	62.50% (95% CI: 38.78%-86.22%)	55.43% (95% CI: 48.25%-62.62%)	10.87% (95% CI: 4.51%-17.23%)	0.19

## 4 Discussion

To our knowledge this is the first study assessing visual capabilities of multimodal GPT in classifying glaucoma using fundus images. We used a benchmark dataset, REFUGE, to test ChatGPT-4 capabilities and compare its accuracy to current available models tested in this dataset. Without performing extra training or fine tuning to the existing model, we assessed its capabilities in assessing glaucoma probability using fundus images. To simulate a real-world scenario where a clinician or user would act based on the initial advice or diagnosis provided by the AI tool, we used the first response generated by ChatGPT-4 for each image, regardless of whether it was accurate or not. ChatGPT-4 had an accuracy of 90% (95% CI 87.06%-92.94%) with high specificity 94.44% (95% CI: 92.08%-96.81%), but relatively low sensitivity 50% (95% CI: 34.51%-65.49%). We also assessed ChatGPT-4 accuracy with other approaches that used REFUGE dataset to classify fundus images into glaucoma/non-glaucoma and reported accuracy metrics, as shown in **Table 4**. The best performance model for each study that tested its model on the REFUGE dataset have been included. While the assessed models achieved superior accuracy, they all have been trained on the same REFUGE dataset training image dataset as part of the model development, which might lead to lower accuracy upon testing in clinical settings, while for the ChatGPT-4, we did not perform any pre-training before the testing (12). The best model we found for glaucoma detection in terms of accuracy on the REFUGE testing dataset was developed by Ganesh et al. (10). They created a novel DL framework named “GD-Ynet” for binary glaucoma classification and optic disc segmentation. Authors modified the basic Ynet architecture by using inception modules instead of convolutional layers. The GD-Ynet model was designed to perform both segmentation and classification tasks within a unified framework.

**Table 4:** Comparison of ChatGPT-4 accuracy against top performances in previous research using the REFUGE Dataset.

Study	Technique	Data for training	Model	Accuracy
Elmoufidi, 2023 (8)	BEMD algorithm (Training set: REFUGE)	ACRIMA and REFUGE	VGG19	99.06
L.K. Singh, 2022 (9)	Cuckoo Search Algorithm (BCS)	ORIGA and REFUGE	SVM	96.23
Ganesh S, 2021 (10)	GD-Ynet	ACRIMA, Drishti-gs, REFUGE, RIGA, and RIM-ONE	Modified U-Net architecture with inception modules	99.50
Sreng S, 2020 (11)	Ensemble method of pretrained deep CNNs as the feature extractors	REFUGE, ACRIMA, ORIGA, RIM-ONE and DRISTI-GS1	Ensemble classifier + SVM	95.75
**Proposed Method**	Assessing the visual capabilities of the multimodal GPT-4 model by combining texts prompts with image inputs.	–	ChatGPT-4	90.00

Previous projects assessed the use of different GPT models in the assessment of text-based case scenarios, for which the GPT model was given textual input to produce convincing textual responses (13). For instance, a recent project by Delsoz et al. assessed the use of ChatGPT-3 to assist in diagnosing glaucoma based on specific clinical case descriptions and compared its accuracy with ophthalmology residents where they found that the accuracy of ChatGPT-3 in diagnosing patients with primary and secondary glaucoma, using specific case examples, was similar or better than senior ophthalmology residents (14). ChatGPT-4 showed superiority in diagnosing complicated cases in other fields of medicine, where a previous study found ChatGPT to have superior diagnostic accuracy in complicated geriatric cases (15).

We identified certain limitations in ChatGPT-4 performance. Specifically, it does not consistently provide identical responses when presented with the same fundus images (i.e., limited reproducibility), which could be related to the “hallucination” problem in its narrative responses (13). The hallucination phenomenon was described in literature as “artificial hallucination”, which is commonly understood as AI generating content that deviates from sense or truth, yet appears to be credible (16, 17). Such hallucinations may lead to wrong diagnoses and improper management. Cai et al. pointed out an example of this with ChatGPT-4 responses to ophthalmology board-style questions, where the model not only provided clinically incorrect answers but also misleading explanations that non-professionals might mistakenly believe to be scientifically true (18). Notably, this behavior was observed during initial experimentations conducted before the onset of the main experimental phase. This stage involved a subset of images that were randomly selected and subjected to multiple presentations to ChatGPT-4. Given the exploratory nature of these preliminary tests, it was not feasible to accurately determine the prevalence of variability across all images in the dataset. Nonetheless, our observations from this phase suggest that a modest proportion of cases within the selected subset exhibited limited reproducibility. Furthermore, ChatGPT-4 occasionally issues apologies and doesn’t perform the required task when asked to provide a medical diagnosis, acknowledging its lack of expertise in the medical field. While this could reduce misuse by the general public, it might restrict physicians’ ability to employ it effectively in healthcare, especially considering the current 40 messages per three hours restriction that OpenAI places on ChatGPT-4 use, as of the time this article was written.

Additionally, since the REFUGE dataset provides high-quality images, it’s important to note that our results may not fully represent the variability in image qualities encountered in clinical practice. This could lead to a reduction in accuracy when applied to images of low quality, which is a common scenario in routine clinical practice. Moreover, considering the class imbalance in the testing dataset (**Figure 1**), F1 might be a valuable measure in these cases. F1 combines precision and recall, where a higher F1 score represent good precision and recall.

The low sensitivity in our study indicates a need for improvement. Therefore, we evaluated the effect of two preprocessing techniques, cropping alone, and cropping in combination with CLAHE. Our findings reveal that cropping alone might enhances the model’s sensitivity in detecting glaucoma, though it seems it does so at the expense of specificity. On the other hand, using CLAHE with cropping yields an improvement in sensitivity compared to unprocessed images. However, this combination does not reach the sensitivity achieved by cropping alone. The optimization of LLMs for specialized tasks such as glaucoma detection from fundus images may require additional fine-tuning with more specialized datasets. The resource investment for such fine-tuning is likely to be lower than the resources required for developing new models from scratch, owing to the pre-existing foundational training of LLMs. Thus, while further research with additional data and resources are necessary to improve ChatGPT-4 in medical diagnostics, its foundational training and versatility in adapting to various tasks suggest that it remains a promising and potentially more resource-efficient solution compared to other AI models

This study explored the capabilities of the recently released multimodal ChatGPT-4 in the assessment of CFPs for glaucoma without pre-training or fine tuning. The importance of this project relates to the assessment of the accuracy of untrained LLMs and what can be achieved compared to existing DL models specifically trained on fundus photographs for this specific task. We found a relatively high accuracy for the ChatGPT-4 model reaching 90% with a specificity of around 94% and a low sensitivity of 50%. The advantage of multimodal ChatGPT-4 is its ability to have more than one input type, which is not the case for other DL models. Future studies should investigate pre-training LLMs on specific medical problems and continue further exploration of the performance and potential applicability to clinics in various settings across different healthcare delivery settings.

## Data availability statement

Publicly available datasets were analyzed in this study. This data can be found here: https://refuge.grand-challenge.org/.

## Author contributions

SA: Conceptualization, Data curation, Investigation, Methodology, Project administration, Supervision, Visualization, Writing – original draft. AM: Conceptualization, Data curation, Formal analysis, Investigation, Methodology, Writing – original draft, Writing – review & editing. MK: Methodology, Supervision, Validation, Writing – review & editing.
