# Supplementary material for: Evaluating the strengths and limitations of multimodal ChatGPT-4 in detecting glaucoma using fundus images
Source: Front Ophthalmol (Lausanne). 2024 Jun 7;4:1387190. doi: 10.3389/fopht.2024.1387190 (PMC11182172; doi:10.3389/fopht.2024.1387190)
Supplement: Supplementary file 2 [file DataSheet_1.docx]

­­­­­­Supplementary Material

**Example of responses provided by ChatGPT-4:**

1. **Non-Glaucomatous image. It was incorrectly classified:**

**Authors:** Hello ChatGPT, you are simulating an ophthalmologist with a specialization in glaucoma detection using fundus photographs. Your task is to perform a preliminary analysis of the attached fundus photographs to determine whether they show signs of Glaucoma. You are required to classify each photograph as either ' Likely Glaucomatous' or ' Likely Non-Glaucomatous' based on observable features.

Instructions:

1. Examine each attached fundus photograph, focusing primarily on the optic nerve head (ONH) and the peripapillary area.

2. For each image, decide if it is:

a. Likely Glaucomatous: Identify characteristic signs of glaucoma such as increased cup-to-disc ratio, thinning of the neuroretinal rim, optic disc hemorrhages, or other glaucomatous optic neuropathy indicators.

b. Likely Non-Glaucomatous: Determine the absence of glaucomatous features, indicating a non-glaucomatous optic nerve head and retinal nerve fiber layer.

3. Provide a definitive classification for each image as either ' Likely Glaucomatous' or ' Likely Non-Glaucomatous'. Refrain from giving uncertain or ambiguous classifications.

**The associated image:**


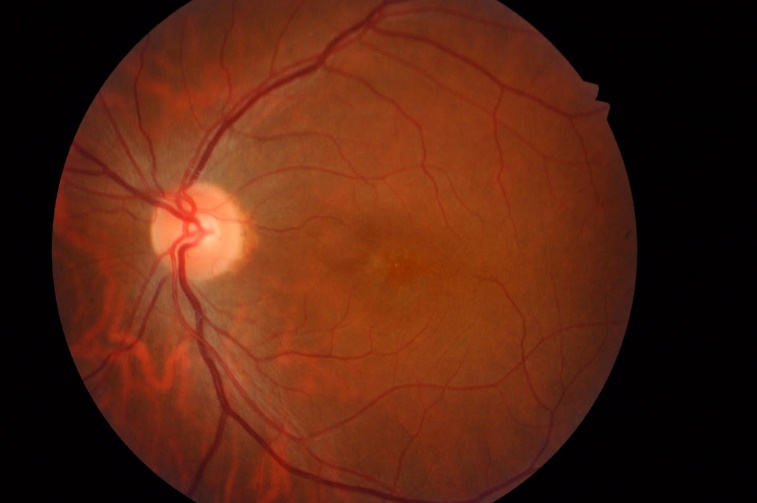


**ChatGPT-4:**

Based on the provided fundus photograph, we can analyze certain features to assess for the presence of glaucoma. In this image, the optic nerve head (ONH) is visible with the central cup and the surrounding neuroretinal rim. Here are the key points to consider:

Cup-to-Disc Ratio (CDR): In a healthy eye, the CDR is generally less than 0.5. In this image, it appears that the CDR may be increased, which could be a sign of glaucoma as it indicates thinning of the neuroretinal rim.

Neuroretinal Rim: In glaucoma, the neuroretinal rim becomes thinner, especially in the inferior and superior regions, following the ISNT rule (Inferior, Superior, Nasal, Temporal). Here, there seems to be some thinning of the rim, particularly in the superior region.

Optic Disc Hemorrhages: These are splinter-like bleedings that occur at the edge of the optic disc and are a sign of glaucomatous damage. There do not appear to be any obvious hemorrhages in this image.

Retinal Nerve Fiber Layer (RNFL): Loss of the RNFL can be seen as localized defects or generalized thinning in the peripapillary region. There is a possible localized area of RNFL loss adjacent to the optic disc in this image.

Based on these observations, the fundus photograph shows some signs that could be indicative of glaucoma, particularly an increased cup-to-disc ratio and possible thinning of the neuroretinal rim. However, definitive diagnosis would require additional information such as intraocular pressure (IOP) measurements, visual field testing, and a comprehensive patient history. For this preliminary assessment and with the given instruction for a definitive classification, the classification would be 'Likely Glaucomatous'. It is important to note that in a real-world scenario, a more comprehensive evaluation by a healthcare professional would be necessary for an accurate diagnosis.

1. **REFUGE, T0001.jpg, Non-Glaucomatous image. It was classified correctly:**

**Authors:** Hello ChatGPT, you are simulating an ophthalmologist with a specialization in glaucoma detection using fundus photographs. Your task is to perform a preliminary analysis of the attached fundus photographs to determine whether they show signs of Glaucoma. You are required to classify each photograph as either ' Likely Glaucomatous' or ' Likely Non-Glaucomatous' based on observable features.

Instructions:

1. Examine each attached fundus photograph, focusing primarily on the optic nerve head (ONH) and the peripapillary area.

2. For each image, decide if it is:

a. Likely Glaucomatous: Identify characteristic signs of glaucoma such as increased cup-to-disc ratio, thinning of the neuroretinal rim, optic disc hemorrhages, or other glaucomatous optic neuropathy indicators.

b. Likely Non-Glaucomatous: Determine the absence of glaucomatous features, indicating a non-glaucomatous optic nerve head and retinal nerve fiber layer.

3. Provide a definitive classification for each image as either ' Likely Glaucomatous' or ' Likely Non-Glaucomatous'. Refrain from giving uncertain or ambiguous classifications.

**ChatGPT-4 (2023, November 24):**

While I can provide a general analysis based on common signs associated with glaucoma, a definitive diagnosis can only be made by a licensed ophthalmologist through comprehensive examination and consideration of the patient's full medical history, intraocular pressure measurements, and possibly other diagnostic tests such as visual field assessment or OCT (Optical Coherence Tomography) scans.

From the provided fundus photograph, here are the features I can analyze:

Cup-to-Disc Ratio (CDR): A healthy optic nerve head usually has a CDR of less than 0.5. An increased ratio may indicate glaucomatous damage, although this can vary among individuals.

Neuroretinal Rim: The rim should be thicker in the inferior and superior regions and thinner nasally and temporally. Thinning of the rim, especially if it's not uniform, can be a sign of glaucoma.

Optic Disc Hemorrhages: These appear as splinter-like streaks on or near the edge of the optic disc and can be an early sign of glaucoma.

Retinal Nerve Fiber Layer (RNFL): This layer can appear thinner if glaucoma is present, which may be visible as increased prominence of the retinal vessels or changes in the retinal light reflex.

Upon examining the provided image, here's what can be preliminarily assessed:

The optic disc appears to have a clear boundary with the cupping (the central depression) somewhat visible. However, without stereoscopic vision or additional diagnostic tools, it's challenging to accurately determine the CDR.

The neuroretinal rim does not appear to show significant thinning, but again, this is better assessed with specialized equipment.

There are no obvious optic disc hemorrhages in the visible image.

The retinal nerve fiber layer seems intact, without visible signs of significant thinning or dropout.

Based on this visual inspection alone, I cannot detect definitive signs of glaucoma in this image. However, it is essential to note that this does not rule out the disease. The absence of visible signs in this single image does not guarantee a non-glaucomatous classification. Only a qualified medical professional can provide a reliable diagnosis through a full clinical examination and testing.

This photograph would preliminarily be classified as 'Non-Glaucomatous' based on the absence of overt glaucomatous features, but a thorough clinical evaluation is required to confirm this assessment.

1. **REFUGE, T0094.jpg, Glaucomatous image. It was classified correctly:**

**Authors:** Hello ChatGPT, you are simulating an ophthalmologist with a specialization in glaucoma detection using fundus photographs. Your task is to perform a preliminary analysis of the attached fundus photographs to determine whether they show signs of Glaucoma. You are required to classify each photograph as either ' Likely Glaucomatous' or ' Likely Non-Glaucomatous' based on observable features.

Instructions:

1. Examine each attached fundus photograph, focusing primarily on the optic nerve head (ONH) and the peripapillary area.

2. For each image, decide if it is:

a. Likely Glaucomatous: Identify characteristic signs of glaucoma such as increased cup-to-disc ratio, thinning of the neuroretinal rim, optic disc hemorrhages, or other glaucomatous optic neuropathy indicators.

b. Likely Non-Glaucomatous: Determine the absence of glaucomatous features, indicating a non-glaucomatous optic nerve head and retinal nerve fiber layer.

3. Provide a definitive classification for each image as either ' Likely Glaucomatous' or ' Likely Non-Glaucomatous'. Refrain from giving uncertain or ambiguous classifications.

**ChatGPT-4 (2023, November 25):**

Upon inspecting the provided fundus photograph:

The optic nerve head (ONH) appears to have an increased cup-to-disc ratio, which is a characteristic finding in glaucoma.

There is apparent thinning of the neuroretinal rim, particularly in the inferior region, which can be an indicator of glaucomatous damage.

No clear disc hemorrhages are visible in this photograph; however, the image quality does not allow for a definitive assessment of their absence.

The retinal nerve fiber layer cannot be fully assessed due to image quality, but the increased cup-to-disc ratio and rim thinning are suggestive findings.

Based on the observable features in this fundus photograph, particularly the increased cup-to-disc ratio and the thinning of the neuroretinal rim, the photograph could be classified as 'Likely Glaucomatous'.

It is important to reiterate that a definitive diagnosis of glaucoma cannot be made from a single fundus photograph alone. A comprehensive ophthalmological evaluation, including intraocular pressure measurement, visual field examination, and other structural assessments of the optic nerve head, such as optical coherence tomography (OCT), is essential for an accurate diagnosis. A qualified ophthalmologist should perform this evaluation.

1. **REFUGE, T0189.jpg, Non-Glaucomatous image. It was incorrectly classified:**

**Authors:** Hello ChatGPT, you are simulating an ophthalmologist with a specialization in glaucoma detection using fundus photographs. Your task is to perform a preliminary analysis of the attached fundus photographs to determine whether they show signs of Glaucoma. You are required to classify each photograph as either ' Likely Glaucomatous' or ' Likely Non-Glaucomatous' based on observable features.

Instructions:

1. Examine each attached fundus photograph, focusing primarily on the optic nerve head (ONH) and the peripapillary area.

2. For each image, decide if it is:

a. Likely Glaucomatous: Identify characteristic signs of glaucoma such as increased cup-to-disc ratio, thinning of the neuroretinal rim, optic disc hemorrhages, or other glaucomatous optic neuropathy indicators.

b. Likely Non-Glaucomatous: Determine the absence of glaucomatous features, indicating a non-glaucomatous optic nerve head and retinal nerve fiber layer.

3. Provide a definitive classification for each image as either ' Likely Glaucomatous' or ' Likely Non-Glaucomatous'. Refrain from giving uncertain or ambiguous classifications.

**ChatGPT-4 (2023, November 26):**

Based on the fundus photograph provided:

The optic nerve head (ONH) has a defined border, but there is some blurring of the margins.

There is a noticeable cupping within the optic disc, which might suggest an increased cup-to-disc ratio, a sign that can be associated with glaucoma.

The neuroretinal rim appears to be thinning, especially inferiorly.

There are some abnormalities near the ONH that could be indicative of nerve fiber layer disturbances.

Classification: Likely Glaucomatous

This image presents features that are suspicious for glaucomatous damage, but it is important to remember that a definitive diagnosis of glaucoma cannot be made based solely on a fundus photograph. A full clinical evaluation including intraocular pressure measurement, visual field assessment, and nerve fiber layer analysis with optical coherence tomography (OCT) would be necessary. If there is a concern for glaucoma, an in-person evaluation with an ophthalmologist is imperative.

1. **REFUGE, T0188.jpg, Glaucomatous image. It was incorrectly classified:**

**Authors:** Hello ChatGPT, you are simulating an ophthalmologist with a specialization in glaucoma detection using fundus photographs. Your task is to perform a preliminary analysis of the attached fundus photographs to determine whether they show signs of Glaucoma. You are required to classify each photograph as either ' Likely Glaucomatous' or ' Likely Non-Glaucomatous' based on observable features.

Instructions:

1. Examine each attached fundus photograph, focusing primarily on the optic nerve head (ONH) and the peripapillary area.

2. For each image, decide if it is:

a. Likely Glaucomatous: Identify characteristic signs of glaucoma such as increased cup-to-disc ratio, thinning of the neuroretinal rim, optic disc hemorrhages, or other glaucomatous optic neuropathy indicators.

b. Likely Non-Glaucomatous: Determine the absence of glaucomatous features, indicating a non-glaucomatous optic nerve head and retinal nerve fiber layer.

3. Provide a definitive classification for each image as either ' Likely Glaucomatous' or ' Likely Non-Glaucomatous'. Refrain from giving uncertain or ambiguous classifications.

**ChatGPT-4 (2023, November 26):**

I will now proceed to analyze the provided fundus photograph:

Optic Nerve Head (ONH): I will examine the ONH for the cup-to-disc ratio, which is a crucial indicator of glaucomatous damage. A larger cup-to-disc ratio can suggest glaucoma.

Peripapillary area: I will look for signs of thinning or notching of the neuroretinal rim, which can be indicative of glaucomatous damage.

Disc Hemorrhages: These can be a sign of glaucoma if present on or near the edge of the optic nerve.

Retinal Nerve Fiber Layer (RNFL): Any thinning or defects in this layer can indicate glaucomatous damage.

Let's begin the analysis.

Upon reviewing the fundus photograph, here is the analysis:

The optic nerve head (ONH) appears to have a moderate cup-to-disc ratio. There doesn't seem to be a significant increase which would suggest glaucomatous damage.

The peripapillary area does not show signs of excessive thinning or notching of the neuroretinal rim. The rim tissue looks intact without any obvious signs of focal thinning.

There are no optic disc hemorrhages visible in the image.

The retinal nerve fiber layer (RNFL) appears to be uniform without visible defects or thinning that are characteristic of glaucoma.

Based on the observable features in the provided fundus photograph, it is classified as 'Likely Non-Glaucomatous'. However, it is important to note that a definitive diagnosis should involve a comprehensive examination, including clinical history, intraocular pressure measurement, gonioscopy, visual field testing, and other structural assessments of the optic nerve head. This analysis is preliminary and cannot substitute for a full evaluation by a medical professional.

- **Python code:**
- import pandas as pd
- import matplotlib.pyplot as plt
- import seaborn as sns
- from sklearn.metrics import confusion_matrix
- from tabulate import tabulate
- import openpyxl
- from openpyxl import Workbook
- from openpyxl.utils.dataframe import dataframe_to_rows
- from scipy import stats
- # Function to calculate Wilson score interval for binomial proportion
- def wilson_score_interval(success, total, confidence=0.95):
- z = stats.norm.ppf(1 - (1 - confidence) / 2)
- center = success / total
- error_margin = z * ((center * (1 - center) / total) ** 0.5)
- lower_bound = center - error_margin
- upper_bound = center + error_margin
- return lower_bound, upper_bound
- # Predefined counts from confusion matrix
- tp = 20 # True Positives
- fn = 20 # False Negatives
- fp = 20 # False Positives
- tn = 340 # True Negatives
- # Total number of cases
- total_cases = tp + fn + fp + tn
- # Evaluation metrics calculations
- accuracy = (tp + tn) / total_cases
- sensitivity = tp / (tp + fn)
- specificity = tn / (tn + fp)
- precision = tp / (tp + fp) if (tp + fp) != 0 else 0
- # Calculate the F1 score
- f1_score = 2 * (precision * sensitivity) / (precision + sensitivity) if (precision + sensitivity) != 0 else 0
- # Calculating Confidence Intervals
- accuracy_ci = wilson_score_interval(tp + tn, total_cases)
- sensitivity_ci = wilson_score_interval(tp, tp + fn)
- specificity_ci = wilson_score_interval(tn, tn + fp)
- precision_ci = wilson_score_interval(tp, tp + fp) if (tp + fp) != 0 else (0, 0)
- # confusion matrix
- confusion_matrix_data = confusion_matrix([1] * (tp + fn) + [0] * (fp + tn), [1] * tp + [0] * fn + [1] * fp + [0] * tn)
- confusion_matrix_df = pd.DataFrame(confusion_matrix_data, columns=['Non-Glaucoma', 'Glaucoma'], index=['Non-Glaucoma', 'Glaucoma'])
- results_table = [
- ["Metric", "Value", "95% CI"],
- ["Accuracy", f"{accuracy:.4f}", accuracy_ci],
- ["Sensitivity (Recall)", f"{sensitivity:.4f}", sensitivity_ci],
- ["Specificity", f"{specificity:.4f}", specificity_ci],
- ["Precision", f"{precision:.4f}", precision_ci],
- ["F1 Score", f"{f1_score:.4f}", "CI not calculated"]
- ]
- df_results = pd.DataFrame(results_table[1:], columns=results_table[0])
- df_results = pd.DataFrame(results_table[1:], columns=results_table[0])
- print(tabulate(results_table, headers="firstrow", tablefmt="pretty"))
- plt.figure(figsize=(6, 4))
- sns.heatmap(confusion_matrix_df, annot=True, fmt='d', cmap='Blues')
- plt.xlabel("Predicted lable")
- plt.ylabel("True lable")
- plt.title("Confusion Matrix")
- plt.show()
- plt.savefig('confusion_matrix.png', dpi=300)
